# Oncologic Outcomes of Thoracoscopic Segmentectomy in Patients with High-Grade Adenocarcinoma Pattern

**DOI:** 10.3390/life15030339

**Published:** 2025-02-21

**Authors:** Stefano Bongiolatti, Alberto Salvicchi, Lavinia Gatteschi, Giovanni Mugnaini, Simone Tombelli, Alessandro Gonfiotti, Luca Voltolini

**Affiliations:** 1 Thoracic Surgery Unit, Careggi University Hospital, 50134 Florence, Italy; stefanobongiolatti@gmail.com (S.B.); giovanni.mugnaini@unifi.it (G.M.); simone.tombelli@unifi.it (S.T.);; 2 Department of Experimental and Clinical Medicine, University of Florence, 50134 Florence, Italy

**Keywords:** VATS segmentectomy, high-grade lung adenocarcinoma, overall survival, recurrence, VATS–lobectomy

## Abstract

Background: Lung adenocarcinoma exhibits heterogeneity among different histological subtypes, with solid and micropapillary subgroups (classified as high-grade) associated with worse prognosis. The aim of this retrospective study was to investigate the impact of high-grade adenocarcinoma on survival in patients undergoing intentional thoracoscopic segmentectomy. Methods: Patients who underwent segmentectomy for clinical-stage IA non-small-cell lung cancer between 2016 and 2023 were reviewed. The adenocarcinoma population was divided and compared based on the presence of high-grade adenocarcinoma >20%, based on the 2021 WHO classification. Survival probabilities were estimated using the Kaplan–Meier method and log-rank test. The Cox proportional hazard regression model was used to test the association between survival and covariates. Results: The adenocarcinoma population included 216 patients, with high-grade adenocarcinoma >20% in 47 (21.7%). A consolidation-to-tumor ratio >0.8 was more frequent in the high-grade adenocarcinoma population. Survival analyses showed that overall (5-year OS rate 57% vs. 90%, *p* < 0.01), cancer-specific (5-year CSS rate 66% vs. 91%, *p* < 0.01) and disease-free survival (5-year DFS rate 53% vs. 75%, *p* < 0.01) were significantly worse in patients with high-grade adenocarcinoma. No significant differences in overall and disease-free survival were observed when compared to a contemporary cohort of lobectomy patients. Recurrence and high-grade pattern (HR 3.26, 95%CI 1.4–7.6, *p* < 0.01) were significant risk factors for reduced overall survival, whereas high-grade adenocarcinoma >20% (HR 2.43, 95%CI 1.25–4.71, *p* < 0.01) and a consolidation-to-tumor ratio >0.8 were risk factors for reduced disease-free survival. Conclusions: The prognosis of high-grade adenocarcinoma is sub-optimal even in radically treated early-stage patients, and close monitoring and a complete bio-molecular assessment should be advisable in light of a multimodal adjuvant approach. However, the different subtypes of adenocarcinoma could be inserted as a staging parameter in future international staging systems.

## 1. Introduction

Lung adenocarcinoma (LADC) is the most frequent histological pattern in the spectrum of non-small-cell lung cancer (NSCLC), with different outcomes based on tumor, node, metastasis (TNM) stage; however, within the same stage, LADC shows heterogeneous results due to the different behaviors of distinct histological patterns. In 2011, the IASLC/ERS/AJCC classification of LADC [1] proposed changes to the 2004 WHO classification, including different histological patterns: solid (SOL), micropapillary (MIP), papillary, acinar and lepidic patterns. These changes were applied in the 2015 WHO classification of invasive non-mucinous LADC [2]. Several prospective and retrospective studies identified SOL and MIP as prognostic factors of poor prognosis [3,4,5,6], whereas lepidic predominant LADC, completely resected, has an excellent prognosis. Based on these data, LADC subtypes have been grouped as grade 1 (low-grade lepidic predominant pattern), grade 2 (intermediate-grade acinar and papillary predominant) and grade 3 (high-grade MIP and SOL) in the latest WHO classification [7]. This three-tiered grading system of non-mucinous LADC is based on a combination of the predominant histological pattern plus the pattern with worst prognosis if it accounts for at least 20% of the tumor [7]. Although the literature is clear about the poor prognosis of MIP and SOL as predominant patterns, some controversies have arisen about the different limits used in the heterogeneous pattern, and the results are not comparable between studies. For example, Bertoglio et al. [8] showed that a percentage of at least >5% of a high-grade LADC pattern was associated with poor outcomes in terms of both overall and disease-free survival in patients surgically treated for LADC pT1-T3N0. On the other hand, Yanagawa et al. showed worse prognoses when MIP and SOL were considered major components (>50%) [9].

The treatment of clinical-stage IA NSCLC has changed following the results of recent randomized controlled trials (RCTs) in which sublobar resection showed an oncological non-inferiority in comparison with lobectomy [10,11,12]. Based on these studies and other large retrospective studies [13,14], some prognostic factors have been identified [10,11,12,13,14], but the influence of the different LADC patterns on survival is not completely understood in this scenario. The aim of this retrospective single-institution study was to investigate the impact of high-grade (HG) LADC patterns at a percentage higher than 20% on survival in a cohort of patients undergoing intentional thoracoscopic segmentectomy (VATS-S) for clinical-stage IA NSCLC.

## 2. Materials and Methods

### 2.1. Patient Population

**Figure 1:**
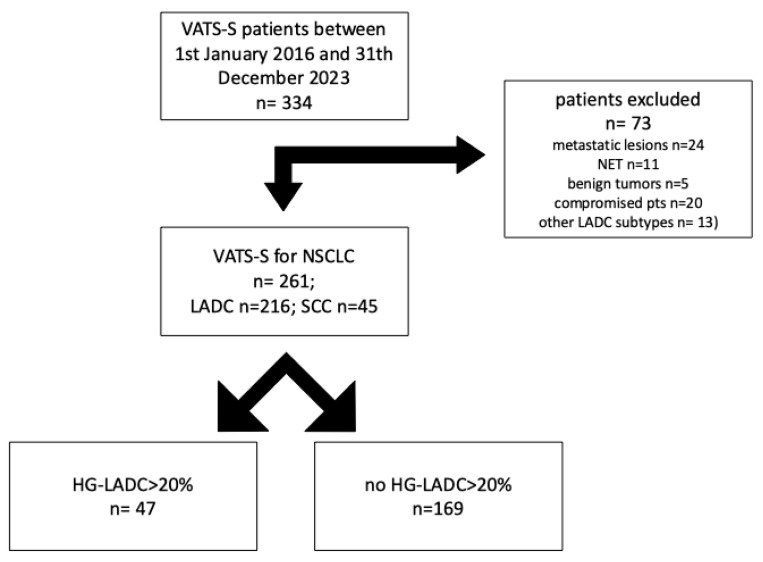
NSCLC cases stratified by High Grade- Lung Adenocarcinoma (HG-LADC) >20%.

Patients who had VATS-S for clinical-stage IA NSCLC from January 2016 to December 2023 at Careggi University Hospital in Florence were retrospectively reviewed. Patients with a diagnosis of benign (n = 5) and neuro-endocrine tumors (n = 11), lung metastases (n = 24), compromised patients (n = 20) and patients affected by mucinous, fetal, enteric-type or colloid LADC (n = 13) were excluded (Figure 1).

Clinical stage was assessed by whole-body computer tomography scan (wb-CT), positron emission tomographic scan (PET-scan), bronchoscopy and, if indicated, endobronchial or esophageal ultrasound (EBUS/EUS) and video-mediastinoscopy to rule out a suspected hilar or mediastinal nodal disease. Clinical and pathological stages were determined according to the American Joint Committee on Cancer 8th Edition TNM Classification [15].

All patients were evaluated by our institutional Multidisciplinary Tumor Board (MTB) and individual treatment was decided on based on clinical stage, patient performance and the most recent international guidelines [16].

In patients fit enough to tolerate a lobectomy, intentional VATS-S with systematic or lobe specific lymph node dissection was proposed for tumors in clinical-stage IA NSCLC with a maximum diameter of 2 cm or smaller and with a peripheral location inside a specific lung segment. For nodules larger than 2 cm or too close to the intersegmental plane, VATS-S with the resection of two or more segments was the preferred procedure. If the surgical margin was less than 2 cm or smaller than the maximum tumor diameter, a frozen section analysis was performed to assess the absence of cancer infiltration [17].

### 2.2. Follow-Up Assessment

Post-operative follow-up was assessed by outpatient visits and included medical history, physical examination and an enhanced contrast wb-CT scan every six months. Recurrence was assessed by radiological findings and was histologically confirmed in every case. Local recurrence was defined as recurrence in the preserved lobe and in the bronchovascular segmental structures; regional recurrence was defined as recurrence in a homolateral lobe/s other than preserved lobe, pleural space, hilar or mediastinal lymph nodes; distant recurrence was defined as any metastasis developed in extra-thoracic organs or contralateral lung.

### 2.3. Ethical Assessment

This retrospective study was reviewed and approved by the Ethics Committee Regione Toscana Area Vasta Centro (approval number 27485, 5 November 2024) and, in accordance with Italian laws for observational retrospective studies, this study granted a waiver of informed consent from study participants.

### 2.4. Statistical Analysis

Statistical analysis was performed using SPSS 24.0 (IBM SPSS Statistics for Macintosh, Version 24.0. Armonk, NY, USA). Standard descriptive statistics were used to summarize data with respect to demographic and oncological characteristics. After the assessment of normality with the Kolgomorov–Smirnov test, continuous variables, expressed as mean values and standard deviations (SDs), were compared using unpaired Student’s *t*-test; non-normally distributed variables were resumed with median and interquartile range and compared with Wilkoxon’s test; categorical variables were analyzed using the χ^2^ test or Fisher’s exact test as appropriate. A two-sided *p*-value below 0.05 was considered statistically significant.

OS was calculated from surgery to death or date of the last follow-up (31 December 2023); CSS was calculated from surgery to death for cancer relapse; DFS was calculated from surgery to the date of the first evidence of recurrence or death. Survival probabilities were estimated using the Kaplan–Meier method and differences were compared with log-rank test. The Cox proportional hazard regression model was used to test the association with OS and DFS and covariates. Selected clinical variables with *p* < 0.10 at univariate analysis were further evaluated by multivariable analysis.

## 3. Results

From January 2016, n = 261 cIA NSCLC patients were treated with intentional VATS-S at our center and analyzed; at pathology, n = 45 (17.2%) patients had squamous-cell carcinoma, whereas n = 216 (83.8%) were operated for LADC. In the LADC group, n = 169 (78.2%) had a low or intermediate LADC pattern, whereas n = 47 (21.7%) had HG-LADC > 20%.

### 3.1. Peri-Operative Results

Demographic, clinical and surgical data are depicted in Table 1. We identified statistical differences in the presence of CTR > 0.8 (*p* < 0.01), pre-operative DLCO% (*p* = 0.015) and the use of simple segmentectomy (*p* = 0.048), as well as a trend toward significance regarding a lower presence of men in the HG-LADC > 20% group (*p* = 0.069). No differences were identified regarding the number of lymph nodes retrieved, margins, pathological stage, upstaging, presence of spread through the air spaces (STAS) and recurrence, as shown in Table 2.

### 3.2. Overall Survival Analysis

At a median follow-up of 36 months, 33 VATS-S patients died of any cause and n = 22 of a cancer-related cause; of these, n = 7 were in the HG-LADC > 20% group.

**Figure 2:**
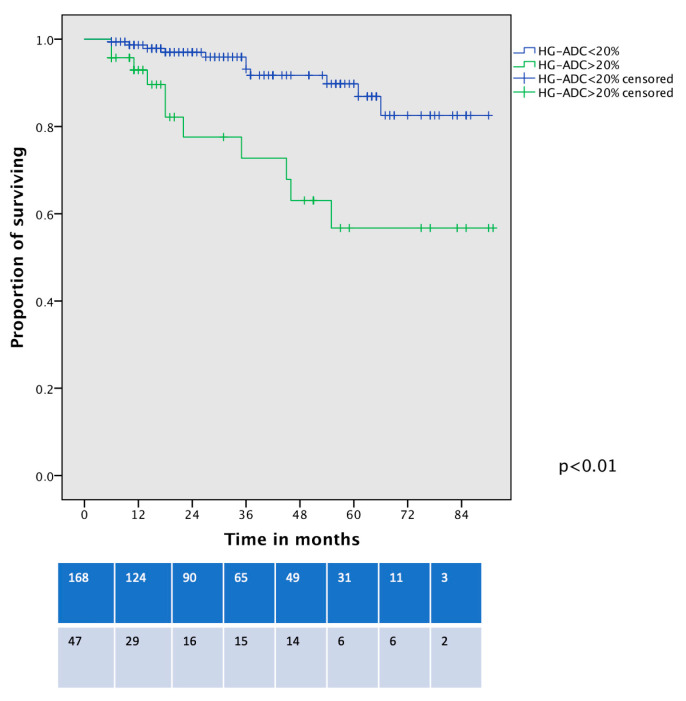
Overall survival curve of VATS-S patients comparing low- and intermediate-grade LADC with HG-LADC > 20%.

**Figure 3:**
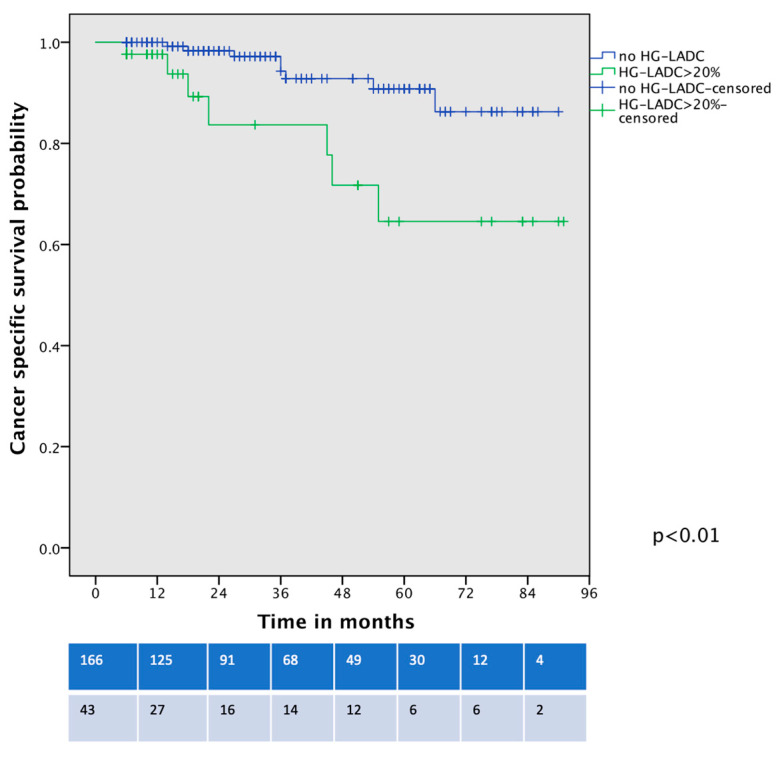
Cancer-specific survival curve of VATS-S patients comparing low- and intermediate-grade LADC with HG-LADC > 20%.

Survival analysis showed that OS was significantly worse between patients with HG-LADC >20% in comparison with low- or intermediate-grade LADC patients who underwent VATS-S (estimated 5-year OS 57% vs. 90%, respectively; all *p* < 0.01, Figure 2). The cancer-specific survival curve (Figure 3) showed significant differences between low- and intermediate-grade LADC in comparison with HG-LADC > 20% (*p* < 0.01). Excluding n = 23 upstaged patients, these significant differences in OS persisted (all *p* < 0.01). Considering only patients with pIA1 and pIA2, significant differences in OS were still present (65% vs. 89%, *p* = 0.018), but no statistical differences were observed regarding CSS (80% vs. 90%, *p* = 0.29).

**Figure 4:**
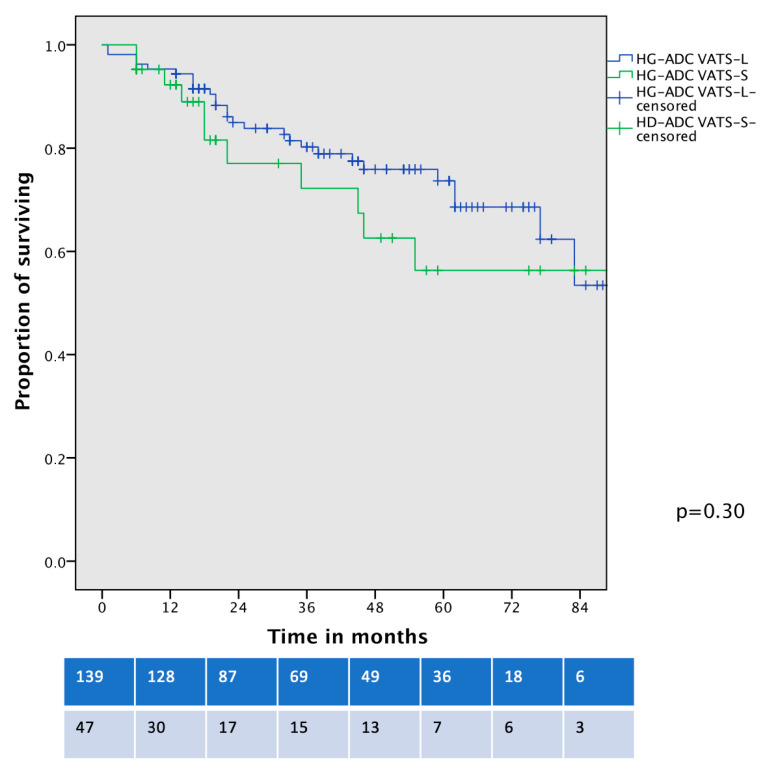
Overall survival curve of cIA HG-LADC > 20% patients undergoing VATS-S or VATS-L.

In a further survival analysis, comparing the OS of contemporary patients with clinical-stage IA HG-LADC >20% who underwent VATS-S and VATS–lobectomy, no significant differences were identified (5-year OS 56% vs. 64%, *p* = 0.30, Figure 4).

Recurrence and the presence of HG-LADC >20% were significantly associated with reduced OS in univariate and multivariable Cox regression analysis (Table 3).

### 3.3. Disease-Free Survival Analysis

**Figure 5:**
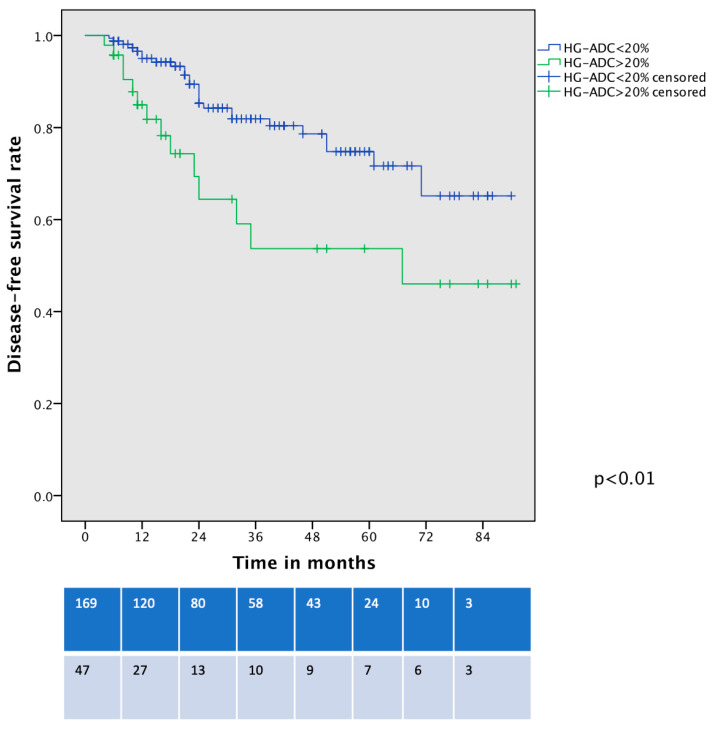
Disease-free survival curve of VATS-S patients comparing low- and intermediate-grade LADC with HG-LADC > 20%.

The recurrence rate was not significantly higher in the HD-LADC group in comparison with low- or intermediate-grade LADC patients, reaching an overall rate of 21.3% vs. 13.6%, *p* = 0.25. The pattern of recurrence was similar among the groups (Table 2). DFS was different between the two groups (53% vs. 75%, *p* < 0.01, Figure 5), and these differences persisted even after the exclusion of n = 23 upstaged patients and cancer-unrelated deaths (*p* < 0.01). Considering only patients with pIA1 and pIA2, the DFS curve showed a trend toward significance of worse DFS for HG-LADC > 20% (5-year DFS 56% vs. 74%, *p* = 0.052). The presence of HG-LADC > 20% and CTR > 0.8 was significantly associated with worse DFS in univariate and multivariable Cox-regression analysis (Table 4).

**Figure 6:**
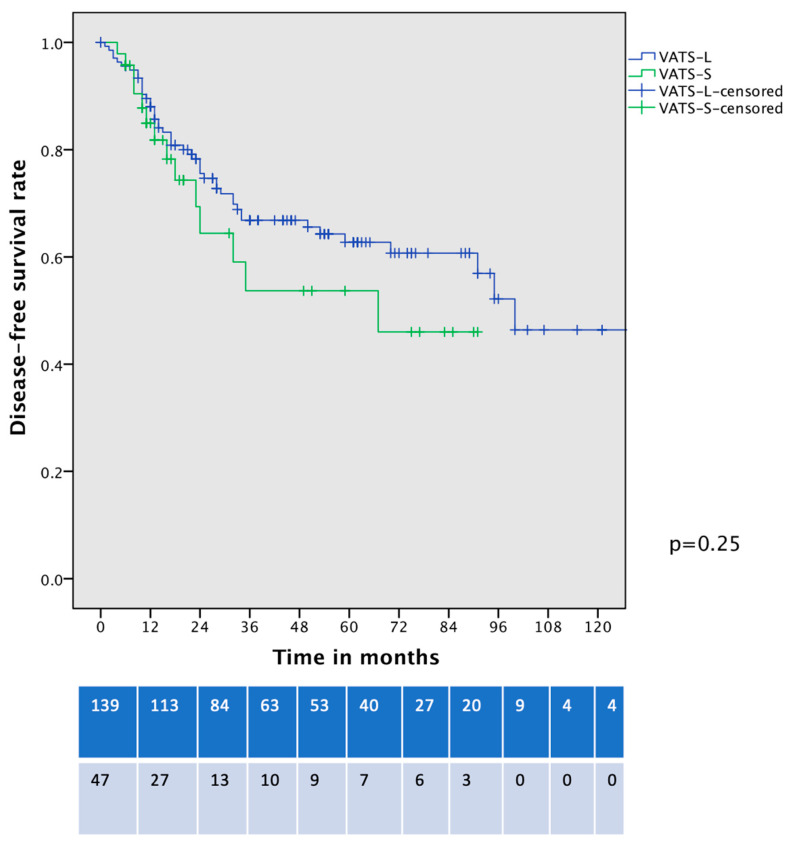
Disease-free survival curve of cIA HG-LADC > 20% patients undergoing VATS-S or VATS-L.

In a comparison of the DFS of contemporary patients with clinical-stage IA HG-LADC > 20% who underwent VATS-S and VATS–lobectomy, no significant differences were identified (5-year DFS 53% vs. 63%, *p* = 0.25, Figure 6).

## 4. Discussion

In the present study, we demonstrated the prognostic relevance of the different LADC patterns and, in particular, we confirmed the poor prognosis of the HG-LADC pattern in a cohort of patient with clinical early-stage adenocarcinoma undergoing VATS-S. OS was significantly worse in patients with HG-LADC > 20% in both clinical-stage IA and in pathological-stage IA1 and IA2, whereas CSS was poorer in the overall population affected by HG-LADC but not in pathological-stage IA1 and IA2. These data confirmed the prognostic role of HG-LADC > 20% as a strong risk factor for VATS-S failure with a deep influence on survival; we can thus assume that the aggressive histological pattern has a great impact on oncological outcomes. Furthermore, DFS was also significantly worse in the HG-LADC > 20% group in the overall population, but a trend toward significance was observed in pathological-stage IA1 and IA2.

The current literature is unanimous in determining the poor prognosis for HG-LADC both in resectable NSCLC (stage I–III) [18,19,20,21] and at early stages such as IA [22]. Unfortunately, some controversies arise from these studies, in particular regarding the percentage of definition of HG-LADC, because some papers classify HG-LADCpl as the presence of MIP and SOL as major components (>50%) [9], while others define it as a percentage of >5% [8] and more recently >20% [7]. Our paper confirmed that the threshold of HG-LADC > 20% has a significant impact on the prognosis of early-stage NSCLC patients undergoing thoracoscopic segmentectomy or lobectomy. Some authors suggest that patients with HG-LADC should undergo a multidisciplinary approach due to the high tendency of HG-LADC to recur and also due to a better prognosis in patients treated with chemotherapy or tyrosine kinase inhibitors (TKIs) in stage IB [22,23]. Unfortunately, in our cohort, few LADCs were completely assessed with bio-molecular and immunohistochemical profile due to the prevalent pathologic early stage, and only upstaged patients were treated with traditional adjuvant chemotherapy and or immunotherapy based on the recent international guidelines. Moreover, the percentage of EGFR was inferior to 10%, as expected for patients from Western countries; also, few patients participated in large-scale RCTs on adjuvant treatment, such as the ADAURA II trial [24].

Another finding is that the prognosis of patients with HG-LADC seems independent of the type of surgery performed, because we did not observe differences in OS and DFS between contemporary populations of VATS-S and VATS-L patients operated for stage IA NSCLC. Other studies comparing DFS between cohorts of lobectomy and sublobar resection patients for clinical-stage IA HG-LADC or a MIP component showed that the recurrence pattern did not differ according to the type of resection [25,26]. In our cohort, patients with HG-LADC underwent simple segmentectomy more frequently, but this finding was not associated with significant differences in survival. These results and our findings corroborate the fact that prognosis is influenced by the biology of LADC and the need for a different pathway of care in these high-risk patients.

Although segmentectomy and lobectomy have similar results in the treatment of the HG-LADC pattern, the clinical prediction of this histological pattern could be crucial to the planning of a more tailored and, if indicated, multimodal approach. Radiomics and the clinical imaging of NSCLC could predict the presence of HG-LADC in some ways and, in particular, HG-LADC seems to be associated with high FGD avidity on PET-CT scan [27] and also seems to be associated with specific CT-findings [27]. In our cohort, CTR > 0.8 was significantly associated with HG-LADC at every percentage, and this is in line with previous published data [27]. Moreover, CTR > 0.8 is significantly associated with worse DFS, and this behavior reflects the more aggressive nature of solid nodules on CT, which are probably the CT representation of HG-LADC. Although PET-CT scans were extensively used in our cohort, SUVmax was not recorded in every case; therefore, we cannot use this parameter to pre-operatively identify HG-LADC, but we noticed a weak association between moderate-to-high FDG avidity with HG-LADC patterns.

This retrospective study has some limitations that should be taken into consideration. First, this is a single-institution study with a limited cohort of patients, and the size of the subgroups with HG-LADC could be considered relatively small. The incidence of spreading through the air spaces (STAS), visceral pleural invasion (VPI), lymphovascular invasion (LVI) and lymph node metastasis was low, and thus, any comparison between these groups and the insertion of these variables into the multivariable model does not seem to be effective. Bio-molecular and immunohistochemical assessments were not obtained for all patients due to the predominance of early-stage NSCLC; therefore, a prognostic prevision based on bio-molecular assessment could not be performed. Other limitations are the length of the post-operative follow-up period, which could be considered relatively short, and also the lack of recurrence data of the VATS-L patients.

In conclusion, the prognosis of HG-LADC is sub-optimal even in radically treated patients at early stages; thus, close monitoring of recurrence and a complete bio-molecular assessment should be advisable in light of a multimodal adjuvant approach in these high-risk patients.

## Figures and Tables

**Table 1:** Demographical, functional and surgical data of HG-LADC > 20% (according to WHO classification) in comparison with low- or intermediate-grade LADC component (ECOG PS: Eastern Cooperative Oncology Group Performance Status; mCCI: modified Charlson Comorbidity Index; ASA: American Society of Anesthesiologists; FEV1: forced expiratory volume in the first second; FVC: forced vital capacity; DLCO: diffusing capacity of the lungs for carbon monoxide; CTR: consolidation-to-tumor ratio; PET: positron emission tomography; cTNM: clinical tumor node metastasis).

Variables	No HG-LADC (n = 169)	HG-LADC > 20% (n = 47)	*p*
Male sex	88 (52.1%)	17 (36.2%)	0.069
Age	72 (14)	72 (12)	0.77
ECOG PS	1 (0)	1 (0)	0.89
mCCI	3 (1)	3 (2)	0.85
ASA	2 (1)	2 (1)	0.59
FEV1%	92.6 (18.7)	88.8 (21.1)	0.29
FVC%	101 (19.6)	101.5 (20.6)	0.91
DLCO%	83.6 (15.7)	76.7 (18.6)	0.015
High or moderate PET avidity	70 (56.9%)	22 (73.3%)	0.14
CTR > 0.8	106 (66.7%)	40 (88.9%)	<0.01
cTNM 1a1 1a2 1a3	30 (17.8%) 100 (59.2%) 39 (23.1%)	11 (23.4%) 22 (46.8%) 14 (29.8%)	0.31
Type of segmentectomy Simple Multiple	83 (49.1%) 66 (39.1%)	31 (66%) 20 (42.6%)	0.048 0.73
Number of segments resected	1 (1)	1 (2)	0.79
Systematic lymph node dissection	153 (90.5%)	42 (89.3%)	0.88

**Table 2:** Post-operative, pathological and recurrence data of HG-LADC > 20% (according to WHO classification) in comparison with low- or intermediate-grade LADC (STAS: spreading through the air space; pTNM: pathological tumor node metastasis).

Variables	No HG-LADC (n = 169)	HG-LADC > 20% (n = 47)	*p*
Number of dissected lymph nodes	9 (5)	10 (7)	0.97
Maximum diameter in mm	17 (8)	18 (10)	0.97
Margins in mm	13 (11)	15 (22)	0.93
pTNM 1a0 1a1 1b0 1b1 1c0 1c1 2a0 2b0 30 40	30 (17.8%) 0 91 (53.8%) 2 (1.2%) 30 (17.8%) 1 (0.6%) 7 (4.1%) 2 (1.2%) 4 (2.4%) 2 (1.2%)	8 (17%) 2 (4.3%) 22 (46.8%) 0 12 (25.5%) 0 1 (2.1%) 1 (2.1%) 0 1 (2.1%)	0.24
STAS	13 (7.7%)	4 (8.9%)	0.76
Upstaged	18 (10.7%)	5 (11.1%)	1
Adjuvant treatment	7 (4.1%)	3 (6.3%)	0.8
Recurrence	23 (13.6%)	10 (21.3%)	0.25
Local recurrence Distant Regional	6 (3.8%) 13 4	2 (4.3%) 6 2	
Major component			
Lepidic	14 (8.2%)		
Acinar	82 (48.5%)	2 (4.2%)	
Papillar	73 (43.1%)	3 (6.3%)	
Micropapillary		7 (14.8%)	
Solid		35 (74.4%)	

**Table 3:** Univariate and multivariable Cox regression analysis on overall survival (OS) (HR: hazard ratio; CI: confidence interval; CTR: consolidation-to-tumor ratio; PET: positron emission tomography; HG-LADC > 20%: high-grade lung adenocarcinoma).

	Univariate Analysis on OS			Multivariable Analysis on OS		
Variable	HR	CI 95%	*p*	HR	CI 95%	*p*
Male sex	1.13	0.45–2.78	0.78			
Age > 70 years	1.33	0.54–3.27	0.52			
CTR > 0.8	4.41	0.58–33.2	0.14			
High–moderate PET avidity	1.12	0.44–2.81	0.81			
Pathological upstaging	2.22	0.84–6.14	0.12			
Tumor size < 2 cm	0.49	0.2–1.2	0.12			
Recurrence	6.13	3.04–12.3	<0.01	7.23	3.54–14.7	<0.01
HG-LADC > 20%	4.2	1.81–9.72	<0.01	3.26	1.4–7.6	<0.01

**Table 4:** Univariate and multivariable Cox regression analysis on disease-free survival (DFS) (HR: hazard ratio; CI: confidence interval; CTR: consolidation-to-tumor ratio; PET: positron emission tomography; HG-LADC: high-grade lung adenocarcinoma).

	Univariate Analysis on DFS			Multivariable Analysis on DFS		
Variable	HR	CI 95%	*p*	HR	CI 95%	*p*
Male sex	1.04	0.6–1.78	0.88			
Age > 70 years	1.29	0.74–2.24	0.36			
CTR > 0.8	5.15	1.16–16.5	<0.01	3.58	1.07–11.9	0.038
High–moderate PET avidity	1.66	0.88–3.12	0.11			
Pathological upstaging	2.61	1.33–5.1	<0.01	1.5	0.62–3.62	0.36
Tumor size < 2 cm	0.74	0.4–1.35	0.33			
HG-LADC > 20%	2.48	1.28–4.78	<0.01	2.43	1.25–4.71	<0.01

## Data Availability

Dataset available on request from the authors.
